# Preparation and Characterization of Size-Controlled Lignin Nanoparticles with Deep Eutectic Solvents by Nanoprecipitation

**DOI:** 10.3390/molecules26010218

**Published:** 2021-01-04

**Authors:** Tong Luo, Chao Wang, Xingxiang Ji, Guihua Yang, Jiachuan Chen, Srinivas Janaswamy, Gaojin Lyu

**Affiliations:** 1 State Key Laboratory of Biobased Material and Green Papermaking, Qilu University of Technology (Shandong Academy of Sciences), Jinan 250353, China; luotongiant@gmail.com (T.L.); jxx@qlu.edu.cn (X.J.); ygh@qlu.edu.cn (G.Y.); chenjc@qlu.edu.cn (J.C.); 2 Department of Dairy and Food Science, South Dakota State University, Brookings, SD 57007, USA; Srinivas.Janaswamy@sdstate.edu

**Keywords:** lignin, nanoparticles, deep eutectic solvents, nanoprecipitation, size-controlled

## Abstract

Lignin nanomaterials have wide application prospects in the fields of cosmetics delivery, energy storage, and environmental governance. In this study, we developed a simple and sustainable synthesis approach to produce uniform lignin nanoparticles (LNPs) by dissolving industrial lignin in deep eutectic solvents (DESs) followed by a self-assembling process. LNPs with high yield could be obtained through nanoprecipitation. The LNPs were characterized by dynamic light scattering (DLS), transmission electron microscopy (TEM), scanning electron microscopy (SEM), Fourier transform infrared spectroscopy (FTIR), and gel permeation chromatography (GPC). Distinct LNPs could be produced by changing the type of DES, lignin sources, pre-dropping lignin concentration, and the pH of the system. Their diameter is in the range of 20–200 nm and they show excellent dispersibility and superior long-term stability. The method of preparing LNPs from lignin–DES with water as an anti-solvent is simple, rapid, and environmentally friendly. The outcome aids to further the advancement of lignin-based nanotechnology.

## 1. Introduction

Lignin is the second most abundant natural biopolymer after cellulose and constitutes one of the major aromatic polymer resources available to mankind [1,2]. Its sustainability and biodegradability coupled with rich functional groups aid significantly in the design and development of a variety of products, e.g., micro- to nano-carriers of bioactive compounds [3,4]. Its limited solubility, however, in organic solvents along with heterogeneous characteristics and random microstructure preclude the downstream processing and valorization [5]. The large particle size and poor water dispersibility further limit its widespread utility [6]. More than 70 million tons of lignin is being produced from several lignocellulose processing plants every year, throughout the world, but are being underutilized and wasted [7,8]. Extending the high value-added applications of industrial lignin, in this regard, is an unmet dream for value addition and economic gain.

A possible choice could be the production of nanoscale lignin, as increased surface area facilitates enhanced chemical and physical interactions of lignin [9,10]. Lignin nanoparticles (LNPs) could be prepared from various molecular sizes of soda lignin by the nanoprecipitation process with tetrahydrofuran (THF) [11]. Similarly, enzymatic hydrolysis of lignin followed by THF dissolution with subsequent self-assembly through water addition results in lignin nanospheres [12]. Another viable protocol is the use of a THF/water system with ultrasonic assistance [13]. Indeed, LNPs display fascinating properties such as favorable dispersibility, high specific surface area, and flexible molecular design [14]. They aid in the preparation of nanostructured materials with several enhanced properties including increased thermal stability, mechanical performance, and barrier properties [15,16,17]. Despite this promising potential, large-scale production of LNPs is far from sufficient. Furthermore, the most commonly used solvent, THF, is volatile, flammable, and difficult to handle, and is not applicable at the industry scale [18]. In this regard, greener solvents that are non-toxic, easy to handle, and recyclable need to be explored in producing LNPs with high yield and controlled size.

Herein, we demonstrate the deep eutectic solvent (DES) as a “green solvent” for the formation of LNPs. Common components of DESs are naturally occurring biocompatible compounds that are not hazardous. Moreover, the synthesis methods of DESs include simple mixing of molecular components, which are economically viable and green chemicals. Such simple preparation, low-cost, low-toxicity, and low-volatility, indeed, gained scientific attention, mainly for the delignification of biomass [19]. Although structural features of lignin isolated during the DES pretreatment have been explored [20,21], its selection as a green solvent to produce nano-lignin has not been investigated comprehensively. Ethanol and THF are common solvents used for LNP preparation, which are hazardous, volatile, flammable, and difficult to handle [22], raising concerns about the safety protocols. Ethanol and THF solubility for industrial lignin is quite limited, too. On the contrary, DES could solubilize around 20–40 wt % of lignin and, thus, offers value addition by reducing the amount of chemical reagent to be used during the LNPs production.

Herein, we propose an effective and environment-friendly approach for preparing the industrial lignin nanoparticles. Our method uses DES as the green solvent through the solvent displacement process. The effect of lignin sources, type of DES, pH of the system, and the concentration of lignin solution on the formation of LNPs were studied. The prepared LNPs were characterized by transmission electron microscopy (TEM), scanning electron microscopy (SEM), dynamic light scattering (DLS), Fourier transform infrared spectroscopy (FTIR), and gel permeation chromatography (GPC). The results reveal size-controlled LNPs with excellent dispersibility and superior long-term stability. The outcome facilitates an inexpensive way of producing LNPs through green protocols and is believed to advance the current lignin-based nanotechnologies with value addition to industrial lignin.

## 2. Results and Discussion

### 2.1. Effects of Different Conditions on Morphology and Size of LNPs

**Figure 1:**
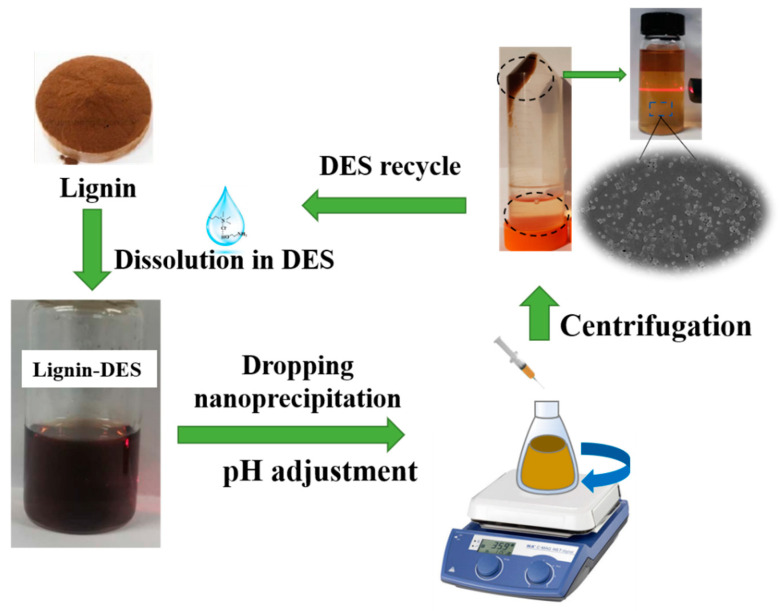
Schematic representation of the preparation procedure of lignin nanoparticles (LNPs). DES: deep eutectic solvent.

LNPs were prepared based on the nanoprecipitation method outlined in Figure 1. The yield, stability, structure, and size of LNPs could be controlled. In order to understand the effects of different conditions, such as the types of DES, the source of lignin, the pre-dropping concentration, and pH on the morphology and size control during nanoprecipitation, the characterization of various LNPs was conducted in detail. The prepared LNPs were coded as x-LNPs-y-z, where x refers to the pre-dropping concentration of lignin solution (3, 6, and 9 wt %), y specifies the DES type, namely, ETA: choline chloride and ethanolamine (ChCl and ETA), EG: choline chloride and ethylene glycol (ChCl and EG), and LA: choline chloride and lactic acid (ChCl and LA) and z corresponds to the pH value of the lignin–DES/water mixture. The LNPs obtained from different conditions are shown in Table 1.

**Figure 2:**
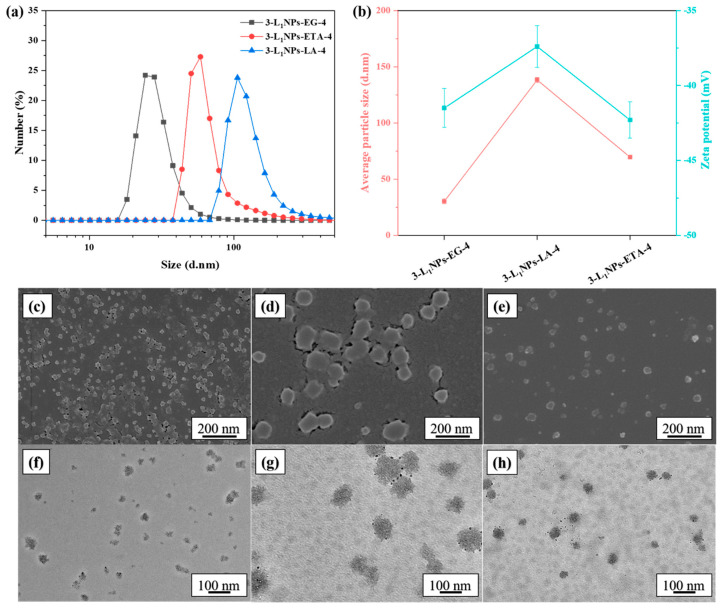
(**a**) Particle size distribution, (**b**) average size and zeta-potential, (**c**–**e**) SEM and (**f**–**h**) TEM images of LNPs obtained with different types of DES. (**c**,**f**) 3-L_1_NPs-EG-4, (**d**,**g**) 3-L_1_NPs-LA-4, (**e**,**h**) 3-L_1_NPs-ETA-4.

**Figure 3:**
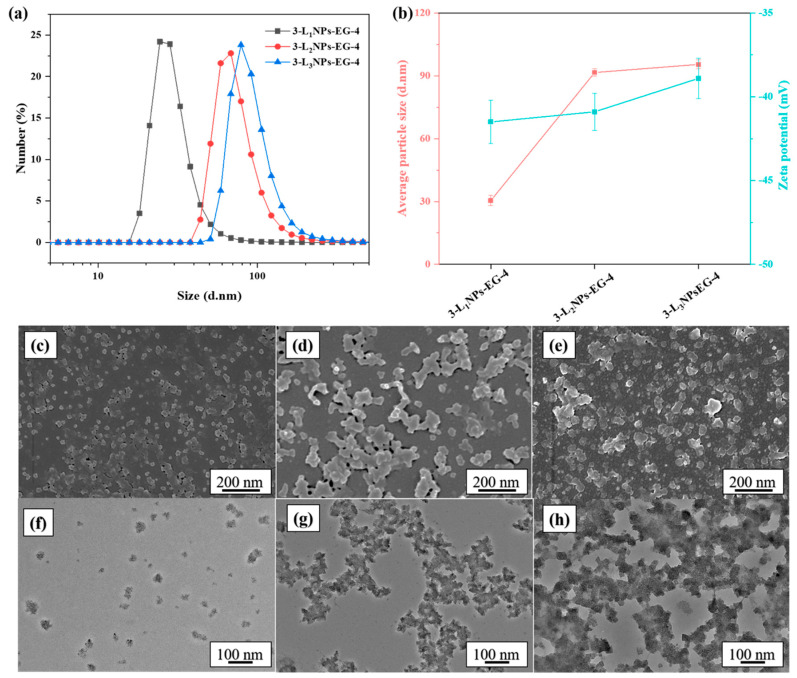
(**a**) Particle size distribution, (**b**) average size and zeta-potential, (**c**–**e**) SEM and (**f**–**h**) TEM images of LNPs obtained from different pristine lignin. (**c**,**f**) 3-L_1_NPs-EG-4, (**d**,**g**) 3-L_2_NPs-EG-4, (**e**,**h**) 3-L_3_NPs-EG-4.

The particle size and morphology of the LNPs are found to be dependent on the DES type (Figure 2). The ChCl and LA and ChCl and ETA yielded an average particle size of 138.2 and 69.7 nm for L_1_, respectively. They are larger than the particle size (30.4 nm) obtained from the ChCl and EG system. Relatively more circular shape particles are characteristics of ChCl and ETA and ChCl and LA, as revealed by the SEM and TEM (Figure 2), in contrast to the ChCl and EG. One possible reason for the non-spherical shapes in the SEM/TEM images may be due to the aggregation of a small number of spherical LNPs during the air drying process [17]. The lignin source also dictates the LNPs size and morphology (Figure 3), and an average size of 91.5 and 95.4 nm is noticed from the L_2_ and L_3_, respectively.

**Figure 4:**
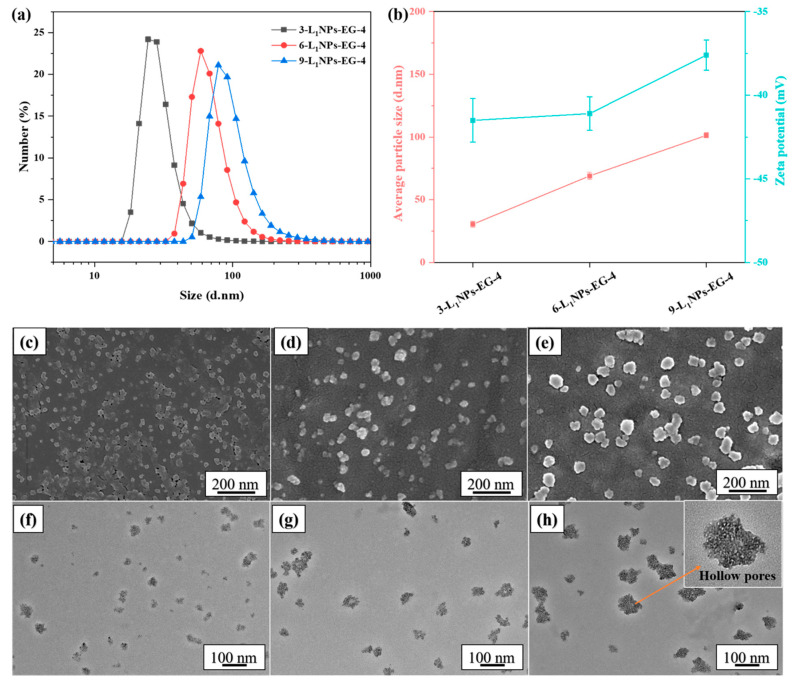
(**a**) Particle size distribution, (**b**) average size and zeta-potential, (**c**–**e**) SEM and (**f**–**h**) TEM images of the LNPs obtained from different pre-dropping concentrations. (**c**,**f**) 3-L_1_NPs-EG-4, (**d**,**g**) 6-L_1_NPs-EG-4, (**e**,**h**) 9-L_1_NPs-EG-4.

Figure 4 shows the effect of the pre-dropping concentration on the morphology and size of LNPs. It appears that pre-dropping concentration will be a significant influencing factor during the production of LNPs. Its increase raises the LNPs’ diameter with the same other conditions, which is consistent with the literature [23,24,25]. The average particle size expands from 30.4 to 101.3 nm (Figure 4a,b) with the pre-dropping lignin concentration of 3 to 9 wt %. Higher amounts might promote more lignin molecules’ participation during nanoparticle formation leading to a larger diameter [24]. The diameter change is in the range of 20–150 nm as confirmed by the SEM (Figure 4c–e) and TEM (Figure 4f–h). The morphology is irregular with hollow pores; such a structure could be advantageous for coatings, drug delivery, and nano-composite materials, to name a few uses.

**Figure 5:**
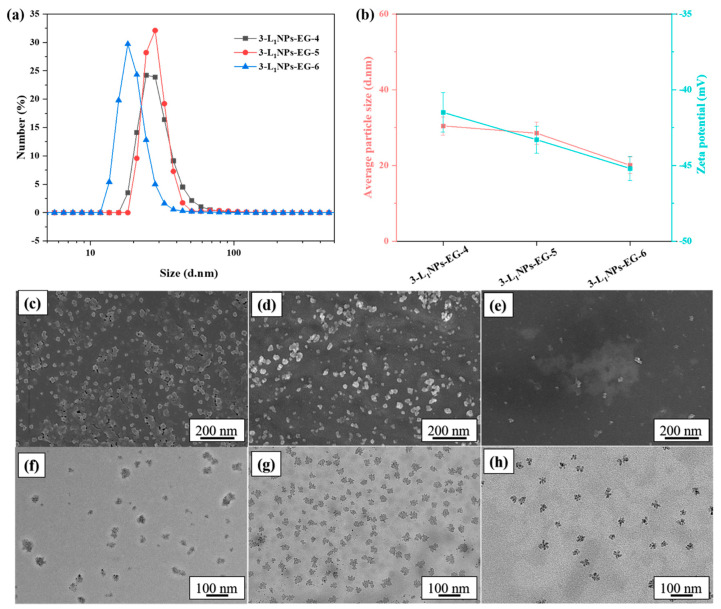
(**a**) Particle size distribution, (**b**) average size and zeta-potential, (**c**–**e**) SEM and (**f**–**h**) TEM images of LNPs obtained at different pH conditions. (**c**,**f**) 3-L_1_NPs-EG-4, (**d**,**g**) 3-L_1_NPs-EG-5, (**e**,**h**) 3-L_1_NPs-EG-6.

Lignin aggregates via stronger electrostatic interactions among its aromatic moieties. Its hydrotropic properties further decrease at acidic and neutral conditions leading to self-assembly of nanoparticles [26,27]. Consequently, the pH of the lignin solution influences the resulting nanoparticle diameter. As the pH rises from 4 to 6, the average size is found to reduce from 30.4 to 20.1 nm (Figure 5). This suggests that LNPs could be dispersed easily at higher pH settings.

### 2.2. Yield and Stability of LNPs

**Figure 6:**
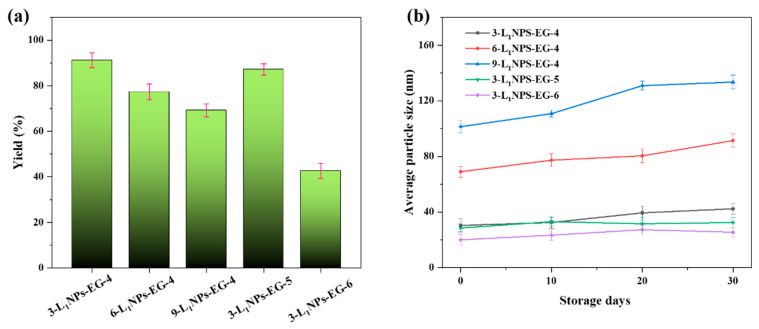
(**a**) The yield of LNPs obtained from different experimental conditions and (**b**) average particle size of the LNP dispersions for 30 days.

The particle size of the LNPs could be controlled easily using our approach. The yield shows a reducing trend when increasing the pre-dropping concentration (Figure 6a). The highest yield of 90.3% is noticed at 3 wt % lignin concentration. The yield decreases with the rise of pH (Figure 6a). For example, the 90.3% yield at pH 4 recedes to 42.3% at pH 6. Though the concentration of lignin used, 3–9 wt %, in our research is much higher than the reported 0.05–2% [28,29,30] in the THF system, the obtained LNPs display uniform particle size. This will certainly solve one of the major issues of industrial lignin that hinges on low dissolution rates in ordinary organic solvents with meager yields [9,26], which could be a significant breakthrough but warrants further investigation.

The long-term stability of nanoparticles is critically important for large-scale applications of nanomaterials [31,32,33]. The zeta potential of the LNPs prepared in this research at various conditions is shown in Figure 2b, Figure 3b, Figure 4b and Figure 5b. The negative zeta potential decreases from −41.1 mV to −37.2 mV with an increase of the pre-dropping lignin concentration from 3 to 9 wt %, indicating the stability of LNPs dispersion lessens subtly. The negative charge of the nanoparticles increases with the pH of the environment (Figure 5b). As the pH value surges from 4 to 6, the negative zeta potential value changes from −37.2 mV to −45 mV. The stability of the LNPs at different pre-dropping concentrations and pH are studied by tracking the size change of the nanoparticles as a function of time. The nanoparticles could be kept intact for more than 30 days in a neutral aqueous medium without any size change (Figure 6b). In addition, there is no particle precipitation barring for the higher pre-drop concentration (9 wt %), indicating excellent stability of the LNPs. This phenomenon could be due to the fact that more LNPs will aggregate when they are left standing for prolonged durations at higher concentrations. A steady nanoparticle dispersion is observed upon re-dispersing the LNPs in water. This could be attributed to the formation of electrical double layers due to surface charge related to the hydroxyl groups and carboxyl groups [26].

### 2.3. Physicochemical Properties of Lignin and the LNPs

**Figure 7:**
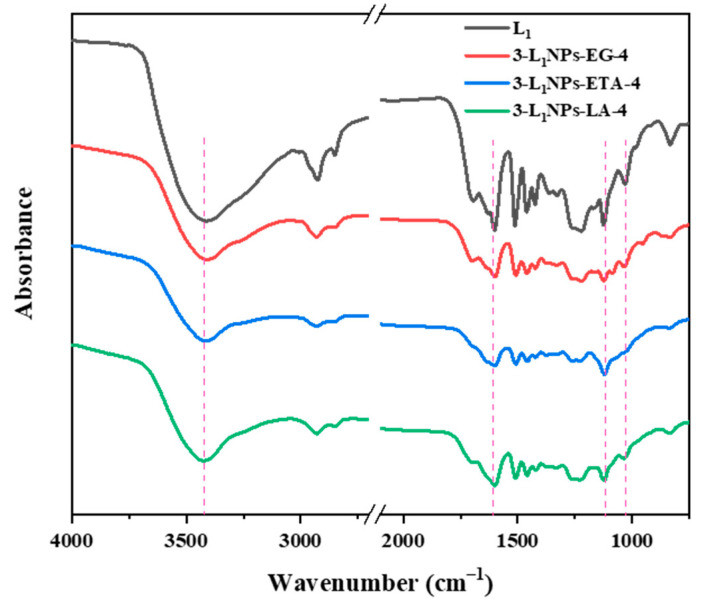
Fourier transform infrared spectroscopy (FTIR) spectra of lignin and LNPs.

With different pristine lignin, the differences in the functional group contents and molecular weights will lead to variable self-assembly processes [34]. In order to compare the chemical structural features of LNPs in the preparation process, FTIR spectra of pristine lignin and the LNPs were analyzed and results are shown in Figure 7. The lignin exhibits bands at 3420 cm^−1^ (hydroxyl groups stretching), 1690 cm^−1^ (C-O stretching), 1599 cm^−1^ (aromatic skeletal vibration), 1509 cm^−1^ (C-C stretching of aromatic skeletal), 1468 cm^−1^ (C-H stretching of aromatic skeletal), 1427 cm^−1^ (aromatic skeletal vibrations combined with C-H in-plane deformation), 1119 cm^−1^ (aromatic C-H deformation of syringyl unit), and 1036 cm^−1^ (OH stretching of primary alcohol) [35,36]. These bands also persist in LNPs obtained from the three DES types without any disappearance or presence of new peaks. It suggests clearly that the preparation process did not impact the initial lignin structure.

The molecular weight of the three pristine lignin samples and LNPs are analyzed by the GPC. The weight-average molecular weight (*M_w_*), number-average molecular weight (*M_n_*), and polydispersity index (PDI) are presented in Table 2. The process of treating lignin with DES at mild conditions, with increasing of the pre-dropping lignin concentration from 3 to 9 wt % and slight decrease in molecular weight and PDI shows that the main structure of the lignin aromatic ring is intact. Interestingly, when increasing pH from 4 to 6, molecular weight swells from 3400 Da to 4200 Da.

### 2.4. Formation Mechanism of LNPs

Generally, the formation of nanoparticles by nanoprecipitation follows the classical nucleation theory (CNT) [37]. The diffusion-limited cluster–cluster aggregation (DLCA) and nuclear growth (NG) are the two significant mechanisms [24,37]. On similar lines, it could be argued as DES and water are miscible, a certain amount of lignin will be able to dissolve in the DES.

**Figure 8:**
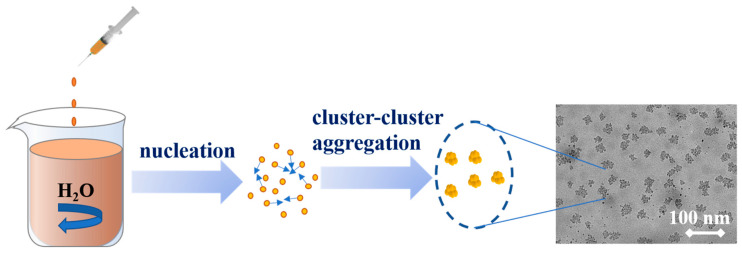
LNPs’ formation mechanism during dropping nanoprecipitation.

Subsequent to the dripping of the lignin–DES solution into water, some highly hydrophobic lignin molecules could form a membrane at the two-phase interface between water and DES, causing water to be entrapped, and forming a balance between the continuous phase and the dispersed phase [9]. The lignin–DES solution will then be wrapped with the membrane. As the reaction time progresses, more and more water molecules penetrate into the membrane, resulting in lignin molecules aggregating on the inner surface of the membrane through layer by layer self-assembly. The resulting cooperative interactions between the hydrophobic lignin chains lead to stable small molecule nanoparticles (Figure 8). The size of the resultant LNPs produced at such a high level of supersaturation could be smaller than 200 nm [9,37]; high concentration lignin solution appears to instantaneously trigger the supersaturation by being injected into the water. Concomitantly, a large number of very small nuclei could be formed that further grow with time.

During this process, DES could be regarded as a surfactant, which is less hydrophobic but still dispersed in the colloidal suspension. Consequently, this unique self-assembly method does not require the use of any other surfactants or crosslinking agents. Overall, the process reported in this research is quite simple and environmentally friendly, and the particle size could be controlled by adjusting the lignin concentrations and solution pH. Further research with more examples of lignin from a variety of sources is needed to fully understand the nanoparticle formation.

## 3. Materials and Methods

### 3.1. Materials

The corncob alkali lignin (L_1_) was obtained from Longlive Bio-Technology Co., Ltd., (Yucheng, China). Kraft lignin (L_2_), isolated from the black liquor of mixed softwood kraft pulping via acid precipitation, was provided by Huatai Paper Co., Ltd. (Rizhao, China). Another commercial alkali lignin (L_3_) was purchased from Tokyo Chemical Industry Co., Ltd. (Tokyo, Japan). Choline chloride (ChCl) was purchased from Yousuo Chemical Technology Co., Ltd. (Shandong, China). Ethanolamine (ETA) was purchased from Aladdin Biochemical Technology Co., Ltd. (Shanghai, China). Ethylene glycol (EG) was purchased from Fuyu Fine Chemical Co., Ltd. (Tianjin, China). Lactic acid (LA) was purchased from Kermel Chemical Reagent Co., Ltd. (Tianjin, China). All chemicals were used as received without further purification unless it was specially mentioned.

### 3.2. Synthesis of DESs

Deep eutectic solvents (DESs) are formed through strong hydrogen bond interactions between suitable hydrogen bond donors (HBDs) and hydrogen bond acceptors (HBAs). DESs are commonly composed of an ammonium salt and a metal halide or a hydrogen bond donor. One of the most widespread HBAs used for the preparation of DESs is choline chloride (ChCl). It can form DESs in combination with HBDs such as urea, carbohydrate-derived polyols, or carboxylic acids. Herein, HBAs of ChCl and HBDs of lactic acid (LA), ethylene glycol (EG), and ethanolamine (ETA) were used for the synthesis of different DESs. The molar ratios of the acidic DES (ChCl and LA), polyol-based DES (ChCl and EG), and the alkaline DES (ChCl and ETA) were 1:9, 1:2, and 1:6, respectively.

### 3.3. Control of Process Parameters for LNP Synthesis

Four parameters were evaluated in the LNPs’ production: pre-dropping concentration, pH, the types of DESs, and the lignin source. Initially, lignin was dissolved in DES at three different concentrations of 3, 6, and 9 wt % (w/w). The solution was magnetic stirred (600 rpm) at room temperature (25 °C) without filtration. Subsequently, the solution was gradually added dropwise into vortexing deionized water at a ratio of 1:20 (solution/water, w/w) with constant stirring (700 rpm) at room temperature. After 30 min, the H_2_SO_4_ and NaOH solution was added until the pH value of the lignin/DES/water mixture reached 4, 5, and 6. The vortexing continued for 1 h and LNPs were formed. Following these steps, the resultant mixtures were then centrifuged at 10,000 rpm for 5 min to precipitate the LNPs. The LNPs were re-dispersed by adding deionized water and the separated liquid was collected through rotary evaporation to recover the DES. The LNPs were freeze dried for subsequent characterization. The yield was determined by measuring the mass of freeze-dried LNPs divided by the mass of the starting lignin.

### 3.4. Characterizations of LNPs

The particle size distribution and average particle size of LNPs in the suspension were detected by dynamic light scattering (DLS) using a Zetasizer instrument (Malvern, Nano-ZS90, Malvern, UK). Number data were collected as a particle size-versus-fraction distribution plot. Scanning electron microscopy (SEM; Hitachi Regulus 8220, Tokyo, Japan) was used to observe the LNPs’ morphology. TEM images were acquired using the JEM-2100F TEM under zero-loss conditions at liquid nitrogen temperature. The FTIR spectra were recorded on a Fourier transform infrared spectrometer (Brooke, ALPHA, Karlsruhe, Germany) operating in the wavelength range of 4000–500 cm^−1^. The molecular weight distribution of the samples was determined by the GPC according to our previous work [38].

## 4. Conclusions

In the study, lignin nanoparticles (LNPs) have been fabricated by an eco-friendly and economic process using a novel lignin–DES system at room temperature. The size-controlled LNPs exhibited excellent dispersibility and superior long-term stability. Increasing the pH and reducing the pre-concentration of lignin decreases the nanoparticle diameter. The chemical structure and molecular weight of LNPs are also preserved. Based on the experimental protocols used in our research, LNPs at a high yield of 90% could be produced, which certainly is promising for the scale-up of LNP production in industry settings. Overall, our study provides a simple and green approach to produce LNPs and we strongly believe that the outcome opens up a window of opportunities for large-scale production of LNPs for applications in controlled release, bioplastics, composites, adsorbents, and dispersants in electro-chemical applications, carbon fibers, and energy storage, to name a few.

## Figures and Tables

**Table 1:** LNPs obtained from different conditions.

Sample	Material	DES System	Pre-Dropping Concentration	pH Value
3-L_1_NPs-EG-4	L_1_	ChCl and EG	3 wt %	4
6-L_1_NPs-EG-4	L_1_	ChCl and EG	6 wt %	4
9-L_1_NPs-EG-4	L_1_	ChCl and EG	9 wt %	4
3-L_1_NPs-EG-5	L_1_	ChCl and EG	3 wt %	5
3-L_1_NPs-EG-6	L_1_	ChCl and EG	3 wt %	6
3-L_1_NPs-ETA-4	L_1_	ChCl and ETA	3 wt %	4
3-L_1_NPs-LA-4	L_1_	ChCl and LA	3 wt %	4
3-L_2_NPs-EG-4	L_2_	ChCl and EG	3 wt %	4
3-L_3_NPs-EG-4	L_3_	ChCl and EG	3 wt %	4

L_1_: corncob alkali lignin, L_2_: softwood kraft lignin, L_3_: commercial alkali lignin, ETA: choline chloride and ethanolamine (ChCl and ETA), EG: choline chloride and ethylene glycol (ChCl and EG), and LA: choline chloride and lactic acid (ChCl and LA).

**Table 2:** Weight-average molecular weight (*M_w_*), number-average molecular weight (*M_n_*), and polydispersity index (PDI) of lignin and LNPs.

Sample	*M_n_*	*M_w_*	PDI
L_1_	1400	3400	2.43
L_2_	1800	3900	2.17
L_3_	1100	2700	2.45
3-L_1_NPs-EG-4	1300	3000	2.31
6-L_1_NPs-EG-4	1600	3400	2.13
9-L_1_NPs-EG-4	1500	3100	2.07
3-L_1_NPs-EG-5	1600	3500	2.19
3-L_1_NPs-EG-6	1300	4200	3.23
3-L_1_NPs-ETA-4	1500	3000	2.00
3-L_1_NPs-LA-4	1400	3000	2.14
3-L_2_NPs-EG-4	1700	3700	2.18
3-L_3_NPs-EG-4	1000	2500	2.50

L_1_: corncob alkali lignin, L_2_: softwood kraft lignin, L_3_: commercial alkali lignin.

## Data Availability

The data presented in this study are available in this article.
